# Pulmonary Actinomycosis: A Diagnostic Challenge

**DOI:** 10.7759/cureus.35118

**Published:** 2023-02-17

**Authors:** Marli Ferreira, Luís Ferreira, Inês Amorim Pereira, André Santos Silva, Inês Henriques Ferreira

**Affiliations:** 1 Internal Medicine, Centro Hospitalar Universitário do Porto, Porto, PRT; 2 Infectious Diseases, Centro Hospitalar Universitário do Porto, Porto, PRT

**Keywords:** actinomyces species, diagnosis, pulmonary tuberculosis, lung cancer, pulmonary actinomycosis

## Abstract

Pulmonary actinomycosis is an uncommon and challenging infectious disease with non-specific symptoms and imaging findings. The authors report a case of a 68-year-old man with diabetes and a history of past smoking who presented with anorexia and weight loss with no significant findings on physical examination. A parenchymal consolidation in the anterior segment of the right upper lobe was detected after a chest computed tomography (CT). Bacterial colonies of Actinomyces species were identified in the histology of transbronchial biopsy. Imaging reassessment after six weeks of treatment with oral amoxicillin showed progression with a high metabolism 10.5 standardized uptake value (SUV) documented on the f-fluorodeoxyglucose positron emission tomography/CT. Concern about the possibility of lung cancer was raised and ruled out by a negative transthoracic needle biopsy. The diagnosis of pulmonary actinomycosis with pyogenic superinfection was presumed. The patient was successfully treated with intravenous amoxicillin and clavulanate for two weeks, followed by six months of oral treatment.

## Introduction

Actinomycosis is an uncommon, chronic, and slowly progressive bacterial infection first described in 1857 [1]. It is caused by the Actinomyces species, a gram-positive bacillus of the human commensal flora of the oropharynx, gastrointestinal, and urogenital tracts. The peak incidence is described in the fourth and fifth decades of life, and males are more often affected (3:1) [2]. Although this disease can affect numerous organs, the most common is cervicofacial actinomycosis, while the pulmonary form is the third most frequent type (approximately 15-20% of all cases) [3].

Pulmonary actinomycosis results primarily from the aspiration of oropharyngeal or gastrointestinal contents. Poor oral hygiene, preexisting dental disease, alcoholism, diabetes, chronic lung disease, or sequelae following tuberculosis are among the risk factors described for this form of the disease [2]. The clinical presentation is diverse, with the most common symptoms being chest pain, productive cough, and dyspnea. With non-specific symptoms and radiological findings, pulmonary actinomycosis is of complex diagnosis. Misdiagnosis with other conditions, such as tuberculosis, lung abscess, or cancer is common [3-5]. This report presents a case of pulmonary actinomycosis with endobronchial involvement.

This article was previously presented as a meeting abstract at the 2022 38º Congresso de Pneumologia on November 10, 2022.

## Case presentation

**Figure 1:**
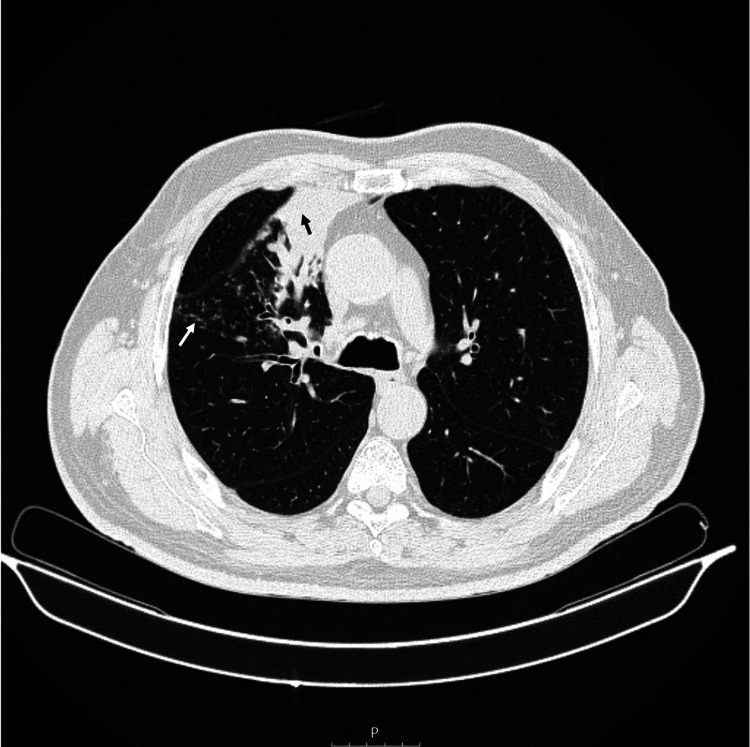
Initial chest computed tomography Chest computed tomography showed an area of parenchymal consolidation in the anterior segment of the upper right lobe of the lung (black arrow) with centrilobular micronodules in a tree-in-bud morphology (white arrow).

A 68-year-old man with a history of type II diabetes, hypertension, overweight, dyslipidemia, gastroparesis, and past smoking (70 pack years) was being evaluated in an outpatient appointment after an episode of community-acquired pneumonia in November 2020, for which he received a seven-day course of amoxicillin and clavulanate. In April 2021, anorexia and weight loss were reported. A control chest CT revealed a parenchymal consolidation in the anterior segment of the upper right lobe of the lung with numerous centrilobular micronodules in a tree-in-bud morphology (Figure 1). At this stage, there were no significant findings on physical examination or respiratory insufficiency/hypoxemia. Laboratory tests showed a white blood cell count of 13 840/uL, hemoglobin levels of 14,4g/dL, C-reactive protein of 130mg/L (0-5mg/L), and an erythrocyte sedimentation rate of 58mm/h. Flexible bronchoscopy revealed stenosis on the anterior segment of the right upper lobe bronchus. Bronchoalveolar lavage cultures were negative. A transbronchial biopsy was performed and despite negative cultures, the histology showed the presence of branched filamentous gram-positive colonies consistent with Actinomyces species.

The diagnosis of pulmonary actinomycosis was presumed. As the patient was being managed as an outpatient without respiratory insufficiency, treatment with oral amoxicillin (1 500mg/day) was chosen. After six weeks of treatment, there were no signs of clinical improvement. Control CT showed a dimensional lesion increase, and c-reactive protein (161mg/L) and erythrocyte sedimentation rate (116mm/h) presented the same ominous tendency. An f-fluorodeoxyglucose (FDG) positron emission tomography/computed tomography (PET/CT) revealed an area of parenchymal consolidation in the upper right lobe with a high metabolism [early maximum standardized uptake value (SUV) of 10.5] and also hilar and mediastinal lymph nodes with SUV between three and four.

**Figure 2:**
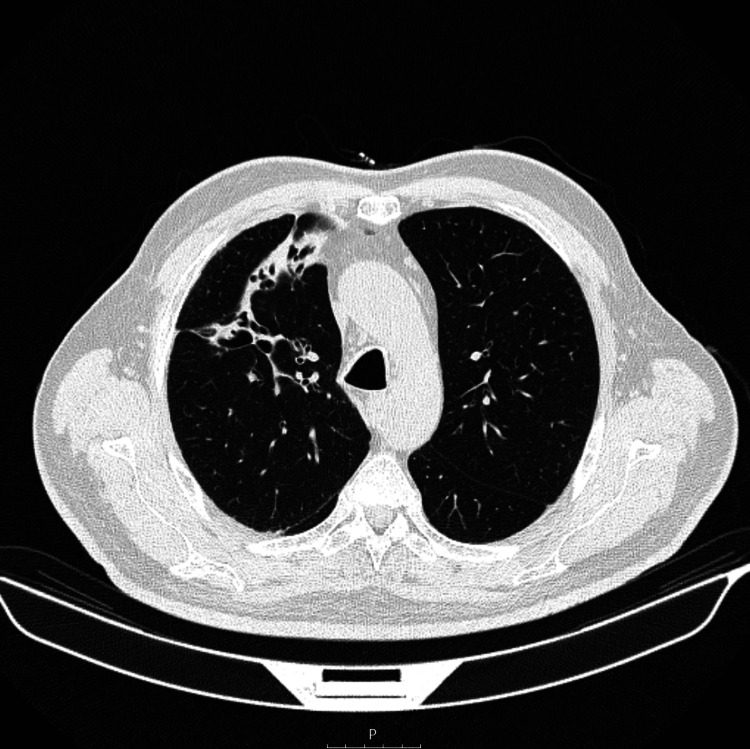
Chest computer tomography after six months of treatment Chest computed tomography reevaluation at the end of the treatment with complete imaging remission, although with sequelae bronchiectasis.

At this point, the results of F-FDG PET/CT combined with the clinical and imaging non-response to antibiotic therapy raised suspicion about the possibility of a lung cancer diagnosis. Therefore, a transthoracic needle biopsy was performed: cultures were negative; histopathologic examination showed an exuberant inflammatory infiltrate with lymphoplasmacytic predominance, no signs of malignancy, and negative staining with periodic acid-Schiff (PAS) and Ziehl-Neelsen. The possibility of inadequate antibiotic management was considered, and treatment was switched to an intravenous administration route. Despite negative cultures, clavulanate was added to cover possible co-pathogens. After two weeks on this antibiotic regimen, there was a complete remission of symptoms and laboratory findings. Following two more weeks of oral antibiotic regimen transition, the patient was discharged and was kept on the same treatment for the following six months. After treatment, there was no relapse of symptoms, and control CT revealed full resolution of the process, although with sequelae bronchiectasis (Figure 2).

## Discussion

Pulmonary actinomycosis is a rare disease, especially in developed countries where dental hygiene improvement and antibiotic treatment initiation before actinomycosis fully develops have probably contributed to a decrease in its incidence. Although it is a rare disease, its diagnosis is vital to avoid misdiagnosis with other chronic pulmonary infections or malignancies with subsequent unnecessary procedures/treatments.

There are no specific clinical manifestations of pulmonary actinomycosis, many of which are shared with other chronic lung infections or malignancies. A myriad of features on CT has been described for this disease (cavitation, pleural effusion, lobar consolidation, ground glass opacification, hilar lymphadenopathy, and pleural thickening). Bronchoscopy features of endobronchial involvement are non-specific (granular thickening, submucosal or exophytic mass), and general PET/CT findings of actinomycosis are intense hypermetabolism as in malignancies (maximum SUV of five, four to 13, seven) [4-8]. Thus, bacterial culture and histological examination of tissue obtained with bronchoscopy, percutaneous biopsy, or surgical resection, allied with clinical and radiological features and the response to antibiotic treatment, are the ground for diagnosis.

Microbiological diagnosis of actinomycosis requires some special considerations: rapid transport of samples to the laboratory, prolonged bacterial culture (five to twenty days), and anaerobic growth conditions, considering the microaerophilic or strict anaerobic character of Actinomyces [9]. Thus, cultures remain sterile in more than 50% of cases due to previous antibiotic therapy, inhibition of Actinomycosis growth by overgrowth of associated bacteria, or inadequate short-term incubation. In contrast, histopathologic evaluation of infected tissue is of great interest, as it is usually more sensitive than cultures. Typical microscopic findings include polymorphonucleates, plasma cells, fibroblasts, necrosis with yellowish sulfur granules, and filamentous colonies of gram-positive pathogens [2,4].

The treatment for pulmonary actinomycosis includes antimicrobial therapy with or without surgery. High doses of intravenous penicillin for two to six weeks (18-24 million units daily), followed by oral penicillin for six to twelve months are the current standard of care [4,6]. Drug resistance is not considered a problem in actinomycosis since Actinomyces species are usually extremely susceptible to beta-lactams, especially penicillin and amoxicillin [2]. The prognosis of the disease is usually favorable when adequately treated, avoiding unnecessary surgery. Shorter courses of antibiotic therapy should be avoided, as these patients are at risk for recurrence or local complications [2,10].

## Conclusions

This case mirrors the difficulties of diagnosing and managing this entity. The lack of recognition of pulmonary actinomycosis in developed countries as a differential diagnosis of long-standing abnormal pulmonary lesions can compromise not only an adequate diagnosis but also the state-of-the-art expertise to best manage this condition. A high degree of clinical suspicion should be kept for this entity and targeted educational efforts should be taken to avoid inadequate treatment management as highlighted in this case.

## References

[1] Mabeza GF, Macfarlane J (2003). Pulmonary actinomycosis. Eur Respir J.

[2] Valour F, Sénéchal A, Dupieux C (2014). Actinomycosis: etiology, clinical features, diagnosis, treatment, and management. Infect Drug Resist.

[3] Kim SR, Jung LY, Oh IJ (2013). Pulmonary actinomycosis during the first decade of 21st century: cases of 94 patients. BMC Infect Dis.

[4] Oikonomidis P, Fousekis F, Kotsaftis P, Pilios I, Dimas D, Giannoulis G (2019). A case of pulmonary actinomycosis presented with endobronchial involvement. Respir Med Case Rep.

[5] Higashi Y, Nakamura S, Ashizawa N (2017). Pulmonary actinomycosis mimicking pulmonary aspergilloma and a brief review of the literature. Intern Med.

[6] Ding X, Sun G, Fei G, Zhou X, Zhou L, Wang R (2018). Pulmonary actinomycosis diagnosed by transbronchoscopic lung biopsy: a case report and literature review. Exp Ther Med.

[7] Martínez-Girón R, Pantanowitz L (2021). Pulmonary actinomycosis: cytomorphological features. Monaldi Arch Chest Dis.

[8] Tanaka S, Araki H, Yoshizako T, Kitagaki H, Isobe T (2020). Pulmonary actinomycosis mimicking pulmonary cancer on fluorine-18 fluorodeoxyglucose PET-CT. Cureus.

[9] Grzywa-Celińska A, Emeryk-Maksymiuk J, Szmygin-Milanowska K, Czekajska-Chehab E, Milanowski J (2017). Pulmonary actinomycosis - the great imitator. Ann Agric Environ Med.

[10] Kolditz M, Bickhardt J, Matthiessen W, Holotiuk O, Höffken G, Koschel D (2009). Medical management of pulmonary actinomycosis: data from 49 consecutive cases. J Antimicrob Chemother.

